# Statins attenuate outgrowth of breast cancer metastases

**DOI:** 10.1038/s41416-018-0267-7

**Published:** 2018-11-07

**Authors:** Colin H. Beckwitt, Amanda M. Clark, Bo Ma, Diana Whaley, Zoltán N. Oltvai, Alan Wells

**Affiliations:** 1 Department of Pathology, University of Pittsburgh, Pittsburgh, PA 15261 USA; 2 University of Pittsburgh Cancer Institute, University of Pittsburgh, Pittsburgh, PA 15261 USA; 3 Pittsburgh VA Health System, Pittsburgh, PA 15213 USA; 4 Department of Computational and Systems Biology, University of Pittsburgh, Pittsburgh, PA 15261 USA; 5 Department of Bioengineering, University of Pittsburgh, Pittsburgh, PA 15261 USA

## Abstract

**Background:**

Metastasis in breast cancer foreshadows mortality, as clinically evident disease is aggressive and generally chemoresistant. Disseminated breast cancer cells often enter a period of dormancy for years to decades before they emerge as detectable cancers. Harboring of these dormant cells is not individually predictable, and available information suggests that these micrometastatic foci cannot be effectively targeted by existing therapies. As such, long-term, relatively non-toxic interventions that prevent metastatic outgrowth would be an advance in treatment. Epidemiological studies have found that statins reduce breast cancer specific mortality but not the incidence of primary cancer. However, the means by which statins reduce mortality without affecting primary tumor development remains unclear.

**Methods:**

We examine statin efficacy against two breast cancer cell lines in models of breast cancer metastasis: a 2D in vitro co-culture model of breast cancer cell interaction with the liver, a 3D ex vivo microphysiological system model of breast cancer metastasis, and two independent mouse models of spontaneous breast cancer metastasis to the lung and liver, respectively.

**Results:**

We demonstrate that statins can directly affect the proliferation of breast cancer cells, specifically at the metastatic site. In a 2D co-culture model of breast cancer cell interaction with the liver, we demonstrate that atorvastatin can directly suppress proliferation of mesenchymal but not epithelial breast cancer cells. Further, in an ex vivo 3D liver microphysiological system of breast cancer metastasis, we found that atorvastatin can block stimulated emergence of dormant breast cancer cells. In two independent models of spontaneous breast cancer metastasis to the liver and to the lung, we find that statins significantly reduce proliferation of the metastatic but not primary tumor cells.

**Conclusions:**

As statins can block metastatic tumor outgrowth, they should be considered for use as long-term adjuvant drugs to delay clinical emergence and decrease mortality in breast cancer patients.

## Introduction

Breast cancer is responsible for the second highest number of female cancer deaths and alone accounts for 30% of all new cancer diagnoses in women.^1^ While localized cancer is effectively treated, with a 5-year survival rate of 99%, the presence of metastatic disease reduces this survival rate to a dismal 27%.^1^ The metastatic cascade is thought to begin with an epithelial to mesenchymal transition (EMT) which is often reversed upon reaching distant sites through a mesenchymal to epithelial reverting transition (MErT).^2,3^ Upon reverting back to an epithelial phenotype, tumor cells can enter a period of dormancy which can last years to decades before cells undergo a secondary EMT and outgrow to form clinically evident metastases.^4^ Unfortunately, at diagnosis, many women likely already harbor micrometastases at distant sites.^5^ Thus, therapies to keep micrometastases in a dormant state and prevent their mortal emergence are desirable to prolong breast cancer survival. Since many standard chemotherapies primarily target dividing tumor cells and are ineffective against quiescent dormant tumor cells, new agents that suppress micrometastatic outgrowth are needed.

Clinical development and implementation of new drugs takes years to decades of study and is very costly. As such, repurposing already FDA-approved drugs with favorable safety profiles may allow for more rapid clinical implementation of effective therapies at low cost.^6,7^ The HMG-CoA Reductase (HMGCR) inhibitors have been clinically used for the treatment of cardiovascular disease for three decades and are well tolerated by the majority of patients.^8,9^ Large retrospective, population-based studies have shown that statins reduce breast cancer mortality^10–12^ without influencing incidence of the primary tumor.^13–15^ These clinical data have been supported by cell and animal studies that suggest statins suppress growth, induce apoptosis, and/or decrease invasiveness of breast cancer cells.^16–21^ However, it remains unclear whether statins can suppress outgrowth of metastases in the context of the metastatic microenvironment.

HMGCR catalyzes the rate-limiting step in cholesterol biosynthesis, which involves conversion of HMG-CoA to mevalonic acid. Important products also produced by this metabolic pathway include farnesyl pyrophosphate (FPP) and geranylgeranyl pyrophosphate (GGPP). These intermediate metabolites play significant roles in prenylation of small GTP-binding signaling proteins including Ras.^22^ Previous studies have shown the pleiotropic effects of statins on cancer and other diseases are primarily a result of a reduction in these prenylating groups.^23,24^ Importantly for breast cancer, signaling pathways downstream of Ras, such as PI3K–Akt and the MAP Kinase cascade, are often implicated in tumor cell growth and suppressed in dormant tumor cells.^4,25,26^ Thus, we hypothesize that statins will selectively target tumor cells primed to emerge from dormancy.

We previously demonstrated that E-cadherin membrane expression—a hallmark of the epithelial reversion that mark dormant micrometastatic tumor cells—was both a marker and mechanism of resistance to statin-mediated growth inhibition.^27^ Herein, we show that atorvastatin can directly suppress growth of tumor cells in in vitro, ex vivo, and in vivo models of breast cancer metastasis. Stimulated outgrowth of dormant breast cancer cells can be significantly suppressed by atorvastatin treatment. Moreover, in two independent mouse models of spontaneous breast cancer metastasis, we show that proliferation of the metastases but not the primary tumor is suppressed with atorvastatin treatment. These data suggest the breast cancer mortality benefit but lack of influence on primary tumor incidence is due to divergent effects of statins on primary and metastatic tumor cells. These findings support considering statins as long-term adjuvants for suppressing outgrowth of micrometastatic breast cancer.

## Materials and methods

### Reagents

Atorvastatin (PHR-1422) and rosuvastatin (SML-1264) were obtained from Sigma Aldrich, USA. Atorvastatin and rosuvastatin were dissolved in dimethyl sulfoxide (DMSO) at a concentration of 50 mM. PD98059 (S1177, SelleckChem) was reconstituted in DMSO at a concentration of 20 mM. LY294002 (S1105, SelleckChem) was reconstituted in DMSO at a concentration of 10 mM. Lipopolysaccharide (LPS, Sigma) and mouse epidermal growth factor (EGF, Corning) were reconstituted in sterile deionized water at concentrations of 1 mg/ml and 50 μg/mL respectively.

### Cell sourcing and cell culture

MCF-7 red fluorescent protein (RFP), MDA-MB-231 RFP, MDA-MB-231 RFP/Ecad, and PC-3 RFP were previously transfected and stably selected.^2^ These cell lines will be referred to in the results and discussion without inclusion of the RFP suffix. DU-145 cell lines DU-L (low E-cadherin) and DU-H (high E-cadherin) were used, as previously described.^28^ All cancer cells were maintained in RPMI 1640, GlutaMax Supplement (Gibco, ThermoFisher) supplemented with 10% heat inactivated fetal bovine serum (HI-FBS, Gemini Bioproducts) and 0.5% penicillin-streptomycin (Gibco, ThermoFisher), henceforth referred to as RPMI. Puromycin (Gibco, ThermoFisher) and G418 (Teknova) were used to maintain expression cassettes, at the following concentrations: 1 μg/mL puromycin (MCF-7 RFP), 5 μg/mL puromycin (MDA-MB-231 RFP), 900 μg/mL G418 (MDA-MB-231 RFP/Ecad and PC-3 RFP). Antibiotic selection media was removed at least 24 h prior to beginning experiments. Primary human hepatocytes and non-parenchymal cells were obtained as isolates from excess pathology specimens at the University of Pittsburgh Medical Center (UPMC) as part of a National Institute of Diabetes and Digestive and Kidney Diseases (NIDDK)-funded Liver Tissue and Cell Distribution System run by Dr. David Gellar and funded by NIH contract #HHSN275201700005C. Hepatocytes were isolated by collagenase perfusion for subsequent distribution to investigators. Non-parenchymal cells were further isolated by density centrifugation using a percoll gradient, as previously described.^29^ Three hepatocyte media were used for experiments. First, William’s E Medium (WE, Life Technologies) supplemented with the Hepatocyte Thawing and Plating Supplement Pack (Life Technologies), which will henceforth referred to as “Plating Media” was used during initial establishment of hepatocyte layer and MPS. Second, William’s E Medium supplemented with the Hepatocyte Maintenance Supplement Pack (Life Technologies), which will henceforth referred to as “Maintenance Media”, was used for the 2D co-culture experiments. Third, Physiologic Hepatocyte Media, made in a base of Williams E Media and containing the following components at final concentrations listed in the subsequent parenthesis: glucose (100 mg/dL), linoleic acid (5.35 µg/mL), GlutaMax Supplement (Thermofisher, 2 mM), HEPES (Thermofisher, 15 mM), penicillin-streptomycin (Gibco, 0.5%), sodium selenite (6.25 ng/mL), human transferrin (6.25 µg/mL), hydrocortisone (550 nM), insulin (800pM), and human serum albumin (25 mg/mL); this was used during the MPS experiments with the cancer cells.

### Statin IC_50_ curve determination

Cancer cells were seeded in 24-well plates at a concentration of 5 × 10^4^ cells/mL in a volume of 500 μL. The next morning, cells were treated with atorvastatin in half log doses between 100 nM and 100 μM. The vehicle control treatment was 0.2% DMSO. After 72 h of treatment, treatment solutions were aspirated and cells were fixed with 3.7% formaldehyde (F79-1, ThermoFisher) for 15 min. After fixation, cells were incubated with 0.5% w/v crystal violet for 10 min and excess dye was removed by copious washing with tap water. The absorbed dye was released with 2% sodium dodecyl sulfate (SDS) and mixed thoroughly before transferring to a 96-well microplate and reading at 560 nm using a Tecan SpectraFluor microplate reader (Tecan US, Durham, NC). Dose–response curves were generated by fitting a standard, four-parameter sigmoid curve to the data.

### Western blotting

MCF-7 RFP, MDA-MB-231 RFP, and MDA-MB-231 RFP/Ecad cancer cells were seeded in 12-well plates at concentrations of 7.5 × 10^4^ and 20 × 10^4^ cells/mL in a volume of 1 mL RPMI. After 48 h of culture in RPMI without any drug treatment, cells were lysed using RIPA buffer supplemented with 1:100 protease inhibitor cocktail V (CalBioChem, USA) and collected into Eppendorf tubes using a cell scraper. The samples were sonicated for 2 s and centrifuged at 13,000 × *g* for 10 min at 4 °C. The supernatant was carefully separated from the pellet into a new tube for sample preparation. Protein concentration was determined by the BCA Protein Assay Kit (Pierce, ThermoFisher Scientific). Samples were prepared and boiled for 5 min prior to loading. Proteins were separated on a 7.5% Tris Bis-acrylamide gel prepared the same day at 96 V (E-C Apparatus Corp., EC-105) until adequate separation was achieved. Samples were transferred onto a nitrocellulose membrane at room temperature at 300 mA for 1.5 h (E-C Apparatus Corp., EC135-90). After transferring, membranes were blocked in 5% w/v non-fat dry milk in tris buffered saline with 0.5% Tween-20 (TBS-T). Membranes were probed with primary antibodies in 5% w/v non-fat dry milk overnight at 4 °C on a rotator. The primary antibodies used were rabbit anti-GAPDH (1:10,000, G9545, Sigma), rabbit anti-E-cadherin (1:1000, 24E10, Cell Signaling), and rabbit anti-vimentin (1:3000, Cat# ab92547, Abcam). After primary incubation, membranes were washed three times for 10 min each in TBS-T. Anti-rabbit IgG (1:5000, Sigma, A9169) was applied at room temperature in 5% w/v non-fat dry milk for 1 h. After secondary incubation, membranes were washed three times for 10 min each in TBS-T. Membranes were incubated with ECL western blotting substrate (Pierce, ThermoFisher Scientific) and photo developed. Western blots were scanned at a resolution of 300 DPI and grayscale bit depth of 16.

### Breast cancer cells mixed culture

MCF-7 and MDA-MB-231 cells each were harvested, washed with PBS, and resuspended in 2 mL serum-free RPMI. CMFDA (Cat# C7025, Thermofisher) was added to the MCF-7 suspension at a final concentration of 5 μM. CMPTX (Cat# C34552) was added to the MB-231 suspension at a final concentration of 5 μM. After 20 min of incubation at 37 °C, 10 mL serum-containing RPMI was added to each tube, cells were respun, counted, and seeded in 12-well plates at a density of 10^5^ cells/mL in a volume of 0.5 mL in RPMI per cell line. The next day, cells were treated with 1 μM, 5 μM, or 20 μM atorvastatin, with 0.04% DMSO serving as the vehicle (0 μM) condition. Images were taken at 0 h and 72 h after treatment for analysis (see “Image Analysis”).

### Hepatocyte—breast cancer co-culture

Primary human hepatocytes (6 × 10^5^) were seeded onto Nunc Thermanox Plastic coverslips (Cat# 174950, ThermoFisher) in 12-well plates on Day 0 in Plating Media. On Day 1, 1000 MDA-MB-231 RFP or 5000 MCF-7 RFP cells were seeded in each well in Maintenance Media. On Day 2, cells were treated with atorvastatin or rosuvastatin, with or without LY294002 or PD98059, for 72 h in Maintenance Media. On the last 24 h of drug treatment, EdU (Cat# E10187, ThermoFisher) was added to a final concentration of 10 µM to detect dividing cells. After 72 h of treatment, treatment solutions were aspirated and cells were fixed with 3.7% formaldehyde (F79-1, ThermoFisher) for 15 min and then changed to PBS before further processing.

### Microphysiological system for Breast Cancer Metastasis in the Liver

**Fig. 1:**
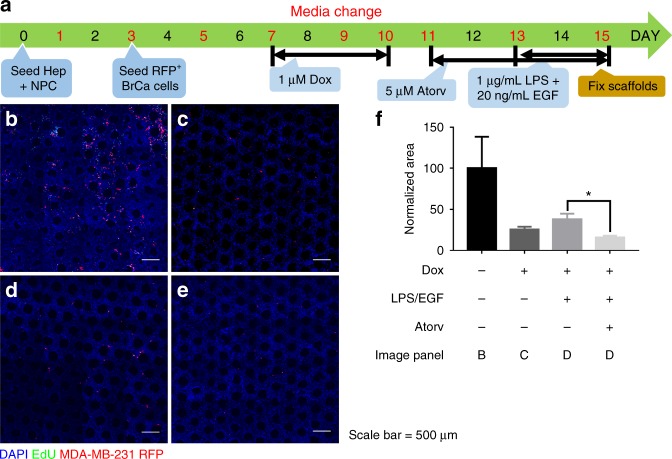
Atorvastatin suppresses stimulated outgrowth of mesenchymal breast cancer cells. The experimental outline for microphysiological system culture of primary human hepatocytes (Hep; 6 × 10^5^), non-parenchymal cells (NPC; 6 × 10^5^), and MDA-MB-231 RFP cells (1000), with media changes represented as red day numbers (**a**). Confocal microscopy was used to generate large Z-stack images for analysis, with representative images shown for each drug treatment: vehicle (**b**), Dox (**c**), Dox + LPS/EGF (**d**), and Dox + LPS/EGF + Atorv (**e**). Red = RFP, Green = EdU, Blue = DAPI. Atorvastatin treatment was able to suppress LPS/EGF stimulated outgrowth of MDA-MB-231 RFP cells (**f**). Dox = 1 µM doxorubicin, LPS/EGF = 1 µg/mL LPS + 20 ng/mL EGF, and Atorv = 5 µM atorvastatin. The data are representative of two independent experiments each of which with a technical *n* = 2. Error bars represent the standard error of the mean (*n* = 4). *p < 0.05. Scalebar = 500 µm

The Liver MPS System (CNBio Innovations) was employed as previously described,^30–33^ and is schematized in Fig. 1a. In brief, 6 × 10^5^ primary human hepatocytes and 6 × 10^5^ primary human non-parenchymal cells were seeded on high impact polystyrene scaffolds coated with 1% rat tail collagen type I (BD Biosciences) on Day 0 in Plating Media. On Day 1, media was changed to Physiologic Media for the remainder of the experiment. On Day 3, 1000 MDA-MB-231 RFP cells were seeded on top of established hepatocyte microtissues. From Day 7–10, scaffolds were treated with 1 µM Doxorubicin (APP Pharmaceuticals LLC). From Day 11–15, scaffolds were treated with 5 µM atorvastatin. Finally, from Day 13–15, scaffolds were treated with 1 µg/mL LPS (Sigma) and 20 ng/mL mouse EGF (Corning). Scaffolds were continuously perfused at a rate of 1 µL/s over the entire culture duration. Media were changed every 2 days and the supernatant was collected for analysis by the University of Pittsburgh Medical Center CLIA-certified clinical labs. On Day 15, the scaffolds were fixed with 2% paraformaldehyde in PBS (Sigma) for 1 h at 4 °C and then changed to PBS before further processing.

### Mice and drug treatments

NOD-SCID gamma (NSG) female mice were obtained from Jackson Labs. Mice were 8 weeks of age upon the start of all experiments. Mice were injected intraperitoneally (IP) with atorvastatin at 2 mg/kg or 10 mg/kg, EdU at 10 mg/kg, or the 2% DMSO vehicle at a volume of 10 μL/mg body weight. Mice were killed by asphyxiation in a chamber with adequate CO_2_ flow as per AVMA procedures.

### Intrasplenic inoculation model for breast cancer metastasis to the liver

On Day 0, mice were anesthetized, their spleens were exposed using a left lateral abdominal incision, and 5 × 10^4^ MDA-MB-231 RFP cells were injected into the spleen in a volume of 50 μL using a 26.5 gauge needle. The peritoneum was closed using one simple stitch with a 5-0 vicryl suture and the skin was closed using staples. Mice were kept on a heating blanket and monitored until they recovered from surgery and were subsequently housed individually. On Day 7, daily IP injections of atorvastatin (at 2 mg/kg or 10 mg/kg) or vehicle were started. On Day 26, two days before euthanizing the mice, a single EdU IP injection was given. On Day 28, mice were euthanized and the spleen, liver, and quad muscle were harvested for analysis. Based on previous studies in our laboratory, the expected rate of liver metastasis (at least micrometastases detected by immunohistochemistry) four weeks after inoculating the tumor cells into the spleen is essentially 100%.

### Mammary fat pad inoculation model for breast cancer metastasis to the lung

On Day 0, 1 × 10^6^ MDA-MB-231 RFP cells were injected into the right inguinal mammary fat pad in a volume of 200 μL using a 26.5 gauge needle and were subsequently housed in cages of two. On Day 10, six IP injections per week of atorvastatin (at 2 mg/kg or 10 mg/kg) or vehicle were started. On Day 30, mice were killed and the mammary tumor and lungs were harvested for analysis.

### Tissue histology

Harvested mouse tissues were immediately fixed in 10% neutral buffered formalin and submitted to the University of Pittsburgh Health Sciences Tissue Bank, where the tissue was paraffin embedded and sectioned. Additionally, Ki-67 and E-cadherin immunohistochemistry (IHC), terminal deoxynucleodityl transferase dUTP nick end labeling (TUNEL), and hematoxylin and eosin (H&E) staining were performed by their well validated protocols.^28^ Blank splenic tumor and liver tumor slides were obtained for in house EdU staining.

### E-cadherin and vimentin immunofluorescence

MCF-7 RFP, MDA-MB-231 RFP, MDA-MB-231 RFP/Ecad, DU-L, and DU-H cells were fixed in 3.7% formaldehyde for 15 min. After fixation, cells were permeabilized with 0.1% Triton-X-100 (ThermoFisher) for 20 min and then blocked in 3% heat shock fraction bovine serum albumin (BSA, Sigma) for 45 min. After blocking, cells were probed using mouse anti-E-cadherin (1:500, Cat# 13-5700, Invitrogen) or rabbit anti-vimentin (1:200, Cat# ab92547, Abcam) overnight at 4 °C in 3% BSA. The appropriate species secondary antibody was used—goat anti-mouse Alexa Fluor 488 (1:200, Cat# A-11001, ThermoFisher) or goat anti-rabbit Alexa Fluor 488 (1:200, Cat# A-11008, ThermoFisher)—for 1 h at room temperature in 3% BSA. After antibody staining, cells were counterstained with 2.5 µg/mL DAPI for 15 min at room temperature and washed three times in normal saline. Coverslips were mounted using a glycerol and PVA-based mounting medium, courtesy of the Center for Biological Imaging at the University of Pittsburgh, and were allowed to harden overnight prior to imaging.

### EdU staining

Cells fixed on coverslips, MPS scaffolds, or mouse tissue slides were permeabilized with 0.1% Triton-X-100 (ThermoFisher) for 20 min then washed once with 3% bovine serum albumin (Sigma). Cells were stained for EdU using the Click-iT EdU Alexa Fluor 488 Imaging Kit (ThermoFisher) per the manufacturer’s instructions. After EdU staining, the cells were counterstained with 2.5 μg/mL (coverslips) or 10 μg/mL (MPS scaffolds) DAPI for 15 minutes at room temperature, and washed three times in normal saline. Coverslips were mounted using a glycerol and PVA-based mounting medium, courtesy of the Center for Biological Imaging at the University of Pittsburgh, and were allowed to harden overnight prior to imaging. MPS scaffolds were kept in PBS until imaging.

### Imaging

**Fig. 2:**
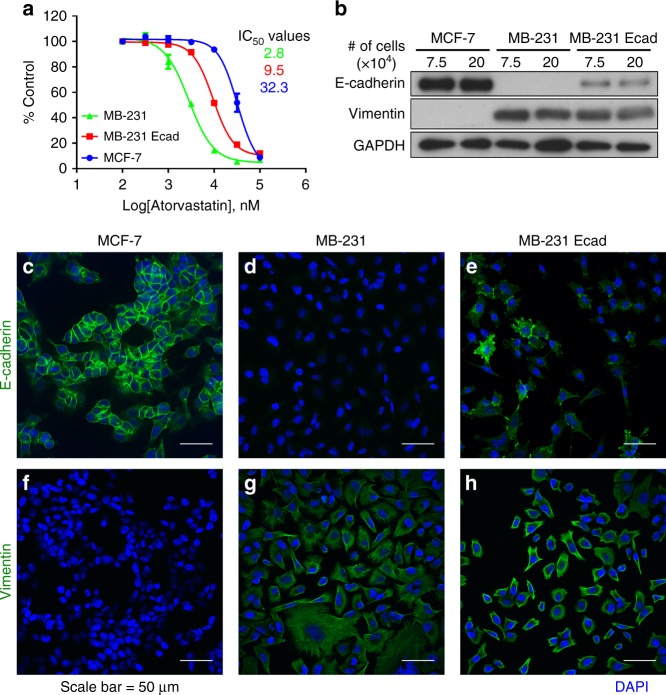
Atorvastatin suppresses mesenchymal breast cancer cells. MCF-7 RFP, MDA-MB-231 RFP, and MDA-MB-231 RFP/Ecad were grown in the presence of atorvastatin for 72 h and total cell number was determined by crystal violet staining and normalized to the control (**a**). IC_50_ values for each cell line are reported (**a**). MCF-7 RFP, MDA-MB-231 RFP, and MDA-MB-231 RFP/Ecad were seeded either at 7.5 × 10^4^ or 20 × 10^4^ cells per well and probed for their total cell levels of E-cadherin and vimentin (**b**). The expression of E-cadherin (**c**–**e**) and vimentin (**f**–**h**) in MCF-7 RFP (**c**, **f**), MDA-MB-231 RFP (**d**, **g**), and MDA-MB-231 RFP/Ecad (**e**, **h**) was probed by immunofluorescent imaging. The data are representative of three independent experiments. Scalebar = 50 µm

**Fig. 3:**
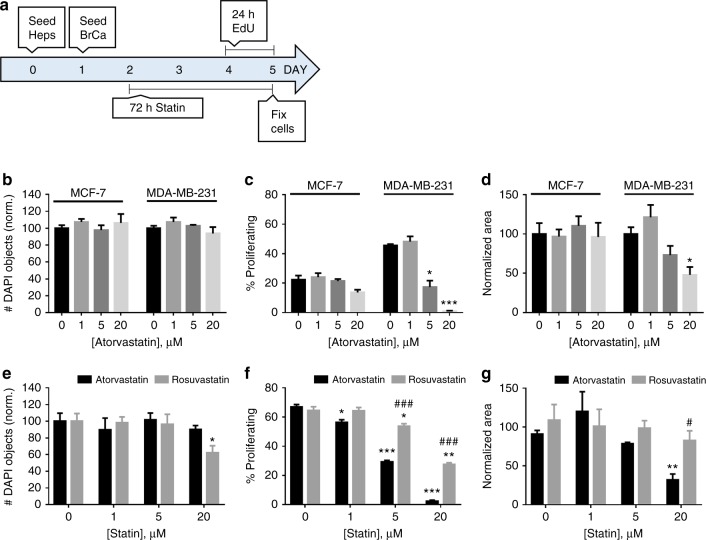
Breast cancer cell line proliferation is more potently suppressed by atorvastatin than rosuvastatin. The experimental outline for hepatocyte—breast cancer co-culture using primary human hepatocytes (Heps; 6 × 10^5^) and RFP-tagged breast cancer cells (BrCa; 5000 MCF-7 RFP or 1000 MDA-MB-231 RFP) (**a**). Atorvastatin treatment in co-cultures with MCF-7 RFP or MDA-MB-231 RFP (**b**–**d**). Atorvastatin did not influence total nuclei number (**b**), but did reduce MDA-MB-231 RFP proliferation (**c**), and total cancer cell area (**d**). Atorvastatin and rosuvastatin treatment in hepatocyte—MDA-MB-231 RFP co-cultures (**e**–**g**). Rosuvastatin demonstrated a reduction in nuclei number at the highest dose (**e**) and a lesser potency at suppressing proliferation (**f**), and total cancer cell area (**g**) than atorvastatin. The data are representative of three independent experiments. Error bars represent the standard error of the mean (*n* = 3). *p < 0.05, **p < 0.01, ***p < 0.001 compared to vehicle. ^#^*p* < 0.05, ^##^*p* < 0.01, ^###^*p* < 0.001 compared to the same dose of atorvastatin

**Fig. 4:**
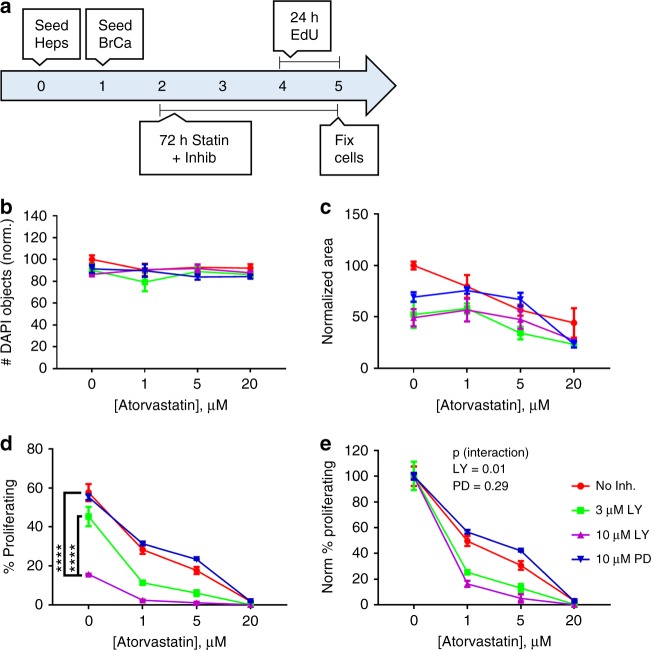
PI3K inhibition potentiates atorvastatin suppression of breast cancer proliferation in co-culture with primary human hepatocytes. The experimental outline for hepatocyte—breast cancer co-culture using primary human hepatocytes (Heps; 6 × 10^5^) and RFP-tagged breast cancer cells (BrCa; 1000 MDA-MB-231 RFP) (**a**). Co-cultures were treated with atorvastatin for 72 h with 3 µM or 10 µM LY294002 (LY; PI3K inhibitor), 10 µM PD98059 (PD; Mek1/2 inhibitor), or vehicle. Nuclei number (**b**) and cancer cell area (**c**) were unaffected by PI3K or Mek1/2 inhibition. PI3K inhibition but not Mek1/2 inhibition significantly inhibited MDA-MB-231 RFP proliferation (**d**) and potentiated the growth suppressive effect of atorvastatin, illustrated by the *p*-value for interaction by two-way ANOVA (**e**). The data are representative of three independent experiments. Error bars represent the standard error of the mean (*n* = 3). The data are representative of three independent experiments. ****p < 0.0001

**Fig. 5:**
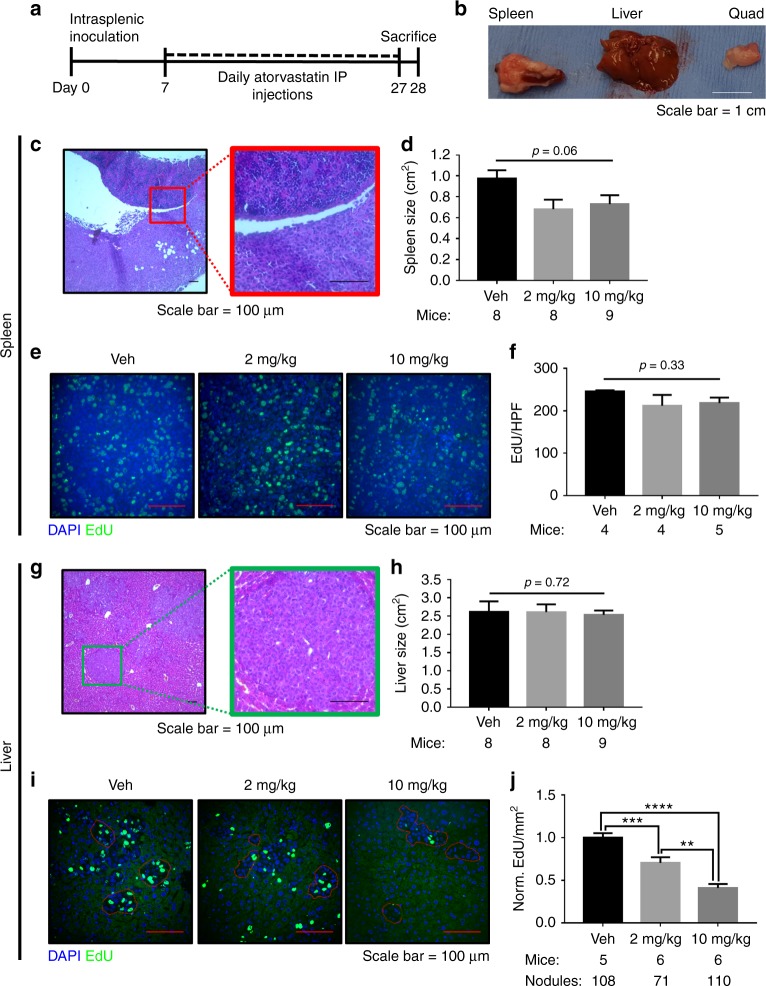
Atorvastatin suppresses breast cancer liver metastatic growth in a splenic mouse model of spontaneous metastasis. The experimental outline for a splenic inoculation model for spontaneous breast cancer metastasis to the liver (**a**). MDA-MB-231 RFP cells (5 × 10^4^) were innoculated into the spleens of mice on day 0, mice were treated with daily intraperitoneal (IP) injections of 2 mg/kg or 10 mg/kg atorvastatin or vehicle (Veh, 2% DMSO in saline) starting on day 7, and mice were euthanized on day 28 for tissue harvesting. Gross pathology was used to examine primary tumor size, the presence or lack of overt liver macrometastases, and myotoxicity (**b**). Histology was determined by H&E staining (**c**) and primary tumor size was determined by splenic cross-sectional area (**d**). Primary tumor proliferation was assessed by EdU incorporation on the 2 days prior to harvesting tissue (**e**, **f**), with EdU represented in green and DAPI counterstaining shown in blue. Histology of liver metastases was determined by H&E staining (**g**) and gross metastatic burden was assessed by liver cross-sectional area (**h**). Liver metastases proliferation was assessed by EdU incorporation on the 2 days prior to harvesting tissue (**i**, **j**), with EdU represented in green and DAPI counterstaining shown in blue. Error bars represent the standard error of the mean. **p < 0.01, ***p < 0.001, ****p < 0.0001. White scalebar = 1 cm, red and black scalebars = 100 µm

**Fig. 6:**
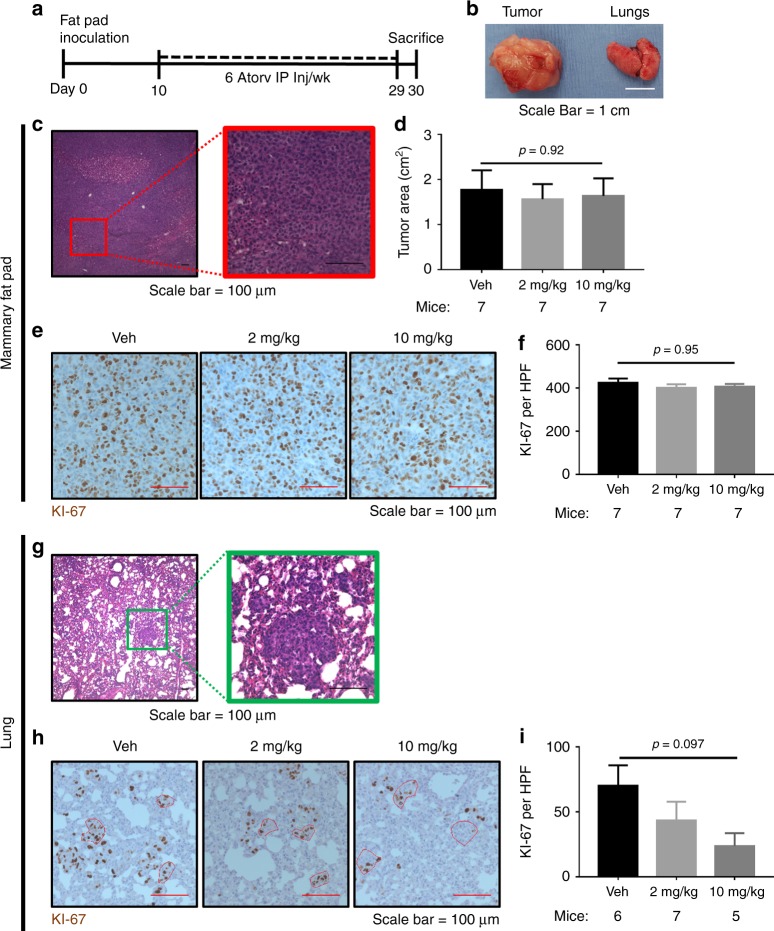
Atorvastatin suppresses breast cancer lung metastatic growth in a mammary mouse model of spontaneous metastasis. The experimental outline for a mammary fat pad (MFP) inoculation model for spontaneous breast cancer metastasis to the lung (**a**). MDA-MB-231 RFP cells (5 × 10^5^) were innoculated into the right MFP of mice on day 0, mice were treated with six per week intraperitoneal (IP) injections of 2 mg/kg or 10 mg/kg atorvastatin or vehicle (Veh, 2% DMSO in saline) starting on day 10, and mice were euthanized on day 28 for tissue harvesting. Gross pathology was used to examine primary tumor size and the presence or lack of overt lung macrometastases (**b**). Primary tumor histology was determined by H&E staining (**c**) and size was determined by tumor cross-sectional area (**d**). Primary tumor proliferation was assessed by Ki-67 immunohistochemistry (**e**, **f**), with Ki-67 represented in brown and counterstained by hematoxolin. Histology of lung metastases was determined by H&E staining (**g**). Lung metastases proliferation was assessed by Ki-67 immunohistochemistry (**h**, **i**), with Ki-67 represented in brown and counterstained by hematoxolin. Significant outliers (*n* = 1 in the vehicle and 10 mg/kg atorvastatin groups) as detected by Grubbs’ test were removed from the analysis in I. Error bars represent the standard error of the mean. White scalebar = 1 cm, red and black scalebars = 100 µm

E-cadherin and vimentin stained cells (Fig. 2) and MPS scaffolds (Fig. 1) were imaged on a Nikon A1 confocal microscope (Nikon) using ×40 and ×10 objectives (Olympus), respectively. For MPS scaffolds, the large image and Z-stack features of the A1 scope were used, to take 4×4 fields of view for each of seven Z-planes, spaced 20 μm apart. The images shown are the maximum intensity projection of the z-stacks whereas the quantification was performed by summation of the individual stacks. For the hepatocyte—breast cancer co-culture coverslips (Figs. 3, 4, S1), cells were imaged on a 90i widefield microscope (Nikon) using a ×10 objective (Olympus). The large image feature was used to take 10 × 14 fields of view (60 mm^2^, 45% coverslip area) for each coverslip. For the widefield images, all 140 fields of view were used for cell counting and quantification. Use of these two scopes was generously provided by the Center for Biological Imaging at the University of Pittsburgh, supported by NIH grant 1S10OD019973-01. Mouse tissue images (Figs. 5, 6, S3, and S5) were taken using an Olympus BX40 upright microscope with a ×10 and ×40 objective using either brightfield (H&E, TUNEL, and Ki-67 IHC images) or fluorescence excitation wavelengths of 405 nm (DAPI) and 488 nm (Click-iT EdU).

### Image analysis

Image analysis was performed in NIS Elements version 5.0. Mixed cancer cells cultures with labeled MCF-7 and MB-231 cells were analyzed using the thresholding function to label all green or red positive area, respectively. All nuclei (breast cancer and hepatocyte) (DAPI) and proliferating nuclei (EdU) channels were labeled using spot detection for bright, clustered spots, and using the same parameters for all images associated with that experiment. The much higher abundance of hepatocytes as compared to cancer cells means the total nuclei number is roughly equivalent to the number of hepatocyte nuclei. The thresholding function was used to label all RFP-positive area (RFP), which represents the area encompassed by the breast cancer cells. Once individual channel masks were created, combined channel masks were generated by using the “having” command, which creates a new mask that illustrates all pixels of the first mask that contain at least one pixel of the second. After generating all masks, data were measured and extracted for analysis. Specifically, the parameters reported are: total nuclei number (DAPI), % Proliferating Cancer Cells ((DAPI + RFP + EdU)/(DAPI + RFP)), and normalized area (percent area of the total image encompassed by the RFP threshold, normalized to the vehicle control).

### Statistics

In all figures, the data are presented as the mean of three independent experiments, when counting the experiments were performed in triplicate, with the error bars representing the standard error of the mean, unless otherwise stated. Comparisons of individual columns in Fig. 3 and Fig. 1 were determined by use of a Student’s two-tailed unequal variance *t*-test. Comparisons of dose curves in Fig. 4 were made using a two-way ANOVA without sample matching, with significance reported as the probability for interaction between the atorvastatin and LY294002 or PD98059 treatment, to test for potentiation. Comparisons in Figs. 5, 6, S1, and S8 were performed using Kruskal–Wallis one-way ANOVA, with post hoc Dunn’s multiple comparison test to determine significance between individual columns. Significance levels are reported in the figure legends and are kept consistent across all figures with symbols denoting *P < 0.05, **P < 0.01, ***P < 0.001, and ****P < 0.0001.

## Results

### Mesenchymal breast cancer cells that lack membrane E-cadherin are more sensitive to atorvastatin suppression

We previously demonstrated that membrane E-cadherin was a marker and mechanism of resistance to atorvastatin treatment, whereas vimentin did not correlate with atorvastatin resistance.^27^ Herein in breast cancer cells, we found the mesenchymal MDA-MB-231 cells were more sensitive to atorvastatin-mediated growth suppression than the epithelial MCF-7 cells (Fig. 2a). As we demonstrated previously,^27^ expression of membrane E-cadherin on MDA-MB-231 cells was sufficient to increase resistance to atorvastatin treatment, though not to the same level as MCF-7 (Fig. 2). To compare epithelial and mesenchymal nature of these cell lines, we determined expression and localization of E-cadherin and vimentin by western blot (Fig. 2b) and immunofluorescent microscopy (Fig. 2c–h). MCF-7 cells expressed E-cadherin to a greater level and with more membrane presentation than MDA-MB-231 Ecad while MDA-MB-231 cells did not express detectable E-cadherin (Fig. 2b–e). In contrast, MCF-7 cells did not express detectable vimentin whereas MDA-MB-231 and MDA-MB-231 Ecad cells expressed vimentin that stained in a filamentous cytoplasmic pattern (Fig. 2b, f–h). When culturing MCF-7 and MDA-MB-231 cells together at the same seeding density, atorvastatin treatment increases the relative abundance of the epithelial MCF-7 by more selectively suppressing growth of the mesenchymal MDA-MB-231 cells (Figure S1). Additionally, atorvastatin treatment does not alter expression of E-cadherin in two DU-145 cell lines with variant E-cadherin expression (Figure S2). Thus, the effects of Atorvastatin on cell viability are deemed independent of the E-cadherin survival effects. These findings confirm our earlier reports^27,34^ of E-cadherin membrane expression correlating with relative resistance to statin growth suppression.

### Atorvastatin suppresses growth of mesenchymal breast cancer cells in co-culture with primary human hepatocytes

The liver is a common site for breast cancer metastasis and is the primary site for drug metabolism in the body.^35^ Moreover, our lab and others have previously shown that co-culture of breast or prostate cancer cells with hepatocytes is sufficient to induce a MErT in the cancer cells.^2,32,36^ Since epithelial cancer cells are less susceptible to atorvastatin treatment, we next wanted to determine whether hepatocytes would influence the efficacy of atorvastatin against breast cancer cells. To accomplish this goal, we co-cultured primary human hepatocytes with MCF-7 RFP or MDA-MB-231 cells for 4 days (Fig. 3a). Importantly, <1% of the cells in these co-cultures were breast cancer cells, in order to model the relative overabundance of liver resident cells as compared to tumor cells in the context of micrometastases.^32^ After allowing the breast cancer cells to integrate into the established hepatocyte monolayer, we treated the co-cultures with atorvastatin for 72 h and included EdU in the culture media for the last 24 h to detect any cells that transited the S phase (Fig. 3a). After treatment, the co-cultures were fixed, imaged using widefield microscopy (Figure S3A), and quantified by image analyses to assess statin toxicity (total nuclei number), influence on breast cancer proliferation (%EdU + cancer cells), and tumor burden (normalized tumor area). Both MCF-7 and MDA-MB-231 cells integrated into the hepatocyte monolayer (Figure S3B,C). Atorvastatin treatment did not affect the total nuclei number (Fig. 3b). While atorvastatin treatment significantly decreased MDA-MB-231 proliferation in a dose-dependent manner, proliferation of MCF-7 cells was unaffected (Fig. 3c). Similarly, atorvastatin decreased tumor burden in co-cultures with MDA-MB-231 cells but not those with MCF-7 cells (Fig. 3d). Atorvastatin appears to be less potent at suppressing growth in this 2D co-culture system than in 2D monoculture of breast cancer cells (Fig. 2). When examining the statin susceptibility of the statin-sensitive prostate cancer cell line, PC-3,^27^ the potency of atorvastatin was similar to that of MDA-MB-231 (Figure S4A). In contrast, atorvastatin was less potent at suppressing the proliferation of the relatively more resistant MDA-MB-231 Ecad cells than was observed with MDA-MB-231 (Figure S4B).

We previously demonstrated that the lipophilic atorvastatin was more potent at suppressing cancer cell growth than the hydrophilic rosuvastatin in cancer cell line monolayer cultures.^34^ To determine whether this effect persisted in the context of the hepatic microenvironment, we co-cultured MDA-MB-231 cells with primary human hepatocytes as previously discussed. In contrast to atorvastatin treatment, we found that rosuvastatin decreased total nuclei number at the highest dose (Fig. 3e) suggesting toxicity of the liver cells. Rosuvastatin was significantly less potent than atorvastatin at all tested doses at suppressing the growth of the MDA-MB-231 cells (Fig. 3f). Thus, while the highest dose of atorvastatin was able to significantly suppress tumor burden, the same dose of rosuvastatin was ineffective (Fig. 3g).

### PI3K inhibition potentiates atorvastatin-mediated suppression of MDA-MB-231 proliferation in co-culture with primary human hepatocytes

The PI3K–Akt and MAP kinase signaling pathways play significant roles in breast cancer cell growth and survival.^25,26^ To determine whether these pathways played a role in breast cancer sensitivity to atorvastatin, we co-cultured MDA-MB-231 cells with primary human hepatocytes, as previously discussed (Fig. 4a). During atorvastatin treatment, cells were additionally treated with 3 µM or 10 µM LY294002 (a pan-PI3K inhibitor) or 10 µM PD98059 (a Mek1/2 inhibitor). Inhibition of PI3K or Mek1/2 did not significantly influence total nuclei number (Fig. 4b) or tumor burden (Fig. 4c) as compared to atorvastatin treatment alone, but PI3K inhibition potentiated atorvastatin-mediated suppression of MDA-MB-231 proliferation in a dose-dependent manner (*p*-value  = 0.01) (Fig. 4d, e). In contrast, Mek1/2 inhibition had no effect on potentiating atorvastatin (Fig. 4d, e). These results are consistent with our finding that, in breast cancer cell lines, atorvastatin decreases Ras prenylation and thus activation.^24^

### Atorvastatin suppresses stimulated outgrowth dormant breast cancer cells

Since we found atorvastatin could decrease the proliferation of breast cancer cells, we next wished to determine whether atorvastatin could suppress stimulated outgrowth of dormant breast cancer cells in the context of the liver microenvironment. To accomplish this goal, we employed an all-human microphysiological system (MPS) for breast cancer metastasis in the liver. This system has been extensively characterized and has been shown to model metastatic dormant-emergent breast cancer, including a transition from epithelial dormant micrometastases to mesenchymal outgrowing macrometastases.^31,32,37^ In this system, a micro-hepatic tissue is established by seeding primary human liver cells, hepatocytes and a full complement of non-parenchymal cells, in a porous scaffold subject to a physiological flow. A full description of the functionality of the MPS has been published.^38,39^ In brief, RFP-labeled breast cancer cells are seeded into these microtissues, perturbed by various drug treatments, and examined 2 weeks later by clinical chemistry assays and microscopy.

The experimental outline is shown in Fig. 1a. In summary, we first treated cells with doxorubicin for 72 h to eliminate cycling cells so that the dormant cell fraction remained. We next treated with atorvastatin for 4 days and on the last two days of treatment, stimulated with an LPS/EGF cocktail, a physiologically relevant inflammatory stimulus designed to drive dormant breast cancer cells to emerge and proliferate.^32^ After 2 days of stimulation, we fixed the cells and imaged scaffolds on a confocal microscope (Fig. 1b–e). We found none of the drug treatments damaged the hepatocytes as shown by no increase in AST and ALT release into the effluent (Figure S5). Atorvastatin significantly suppressed LPS/EGF stimulated outgrowth of the mesenchymal MDA-MB-231 cells (Fig. 1f).

### Atorvastatin suppresses proliferation of breast cancer liver metastases but not the primary tumor

Since we found atorvastatin was able to suppress the stimulated outgrowth of breast cancer cells in the liver MPS, we wanted to examine whether systemic atorvastatin administration in a mouse model for spontaneous breast cancer metastasis to the liver would show the same affect. We have previously demonstrated this model reproduces primary tumor EMT and metastatic tumor MErT.^2,40^ The experimental outline is shown in Fig. 5a. In summary, we inoculated MDA-MB-231 into the spleens of female NSG mice. After allowing the primary tumor to establish for 1 week, we treated the mice with daily IP injections of 2 mg/kg atorvastatin, 10 mg/kg atorvastatin, or vehicle for 3 weeks. Two days before killing the mice, we also injected 10 mg/kg EdU to detect cell proliferation. We found that all mice developed primary tumors in the spleen without having overt liver macrometastases. To assess statin toxicity we examined skeletal muscle; all mice exhibited healthy quadriceps (quad) muscle tissue (Fig. 5b).

We found the primary tumor size did not significantly change with atorvastatin treatment (Fig. 5d). Primary tumors grew alongside the splenic parenchyma and would often invade through the splenic capsule as they grew (Fig. 5c). These primary tumors were marked by a lack of E-cadherin expression (Figure S7A,B) and minimal TUNEL staining (Figure S7A,B). Vimentin expression was not assessed since we previously found its expression to not influence atorvastatin sensitivity.^27^ Primary tumor proliferation was quantified by assessing the EdU positivity of the peripheral zone of the tumor. The proliferation of the primary tumor was not significantly affected by atorvastatin treatment (Fig. 5e, f). Additionally, atorvastatin treatment did not affect TUNEL staining in the primary tumor (Figure S7E).

We found that the total size of the liver did not significantly change with atorvastatin treatment (Fig. 5h). Breast cancer metastases ranged in size, but were at the largest several hundred micrometers in diameter (Fig. 5g); these nodules still represented only a minor fraction of total liver tissue reflecting the micrometastatic nature of the tumor nodules. While small micrometastases exhibited membrane E-cadherin expression, larger metastases (>100 µm) were largely E-cadherin negative (Figure S6C,D), having undergone a second epithelial to mesenchymal transition (EMT).^3,40^ Moreover, liver metastases stained negative for TUNEL (Figure S7C,D). To assess proliferation of liver metastases, EdU staining was performed. Tumor nodules were easy to identify by the lack of green hepatocyte auto-fluorescence (Fig. 5i). We found that atorvastatin significantly decreased proliferation density of metastatic nodules in a dose-dependent manner (Fig. 5j). Similar to the primary tumor, atorvastatin treatment did not affect TUNEL staining in liver metastases (Figure S7F), suggesting that cytotoxic effects were minimal.

### Atorvastatin suppresses proliferation of breast cancer lung metastases but not the primary tumor

Since we found atorvastatin could significantly suppress the growth of micrometastases, we next wanted to determine if atorvastatin would reduce metastatic proliferation at a different site: the lung, the most common site for clinically evident breast cancer metastasis.^41^ The experimental outline is shown in Fig. 6a. In summary, we inoculated MDA-MB-231 breast cancer cells into the inguinal mammary fat pad (MFP) of female NSG mice. After allowing the primary tumor to establish for 10 days, we treated the mice with six IP injections per week of 2 mg/kg or 10 mg/kg atorvastatin or vehicle for 3 weeks. We found that all mice developed primary tumors and were void of overt lung macrometastases (Fig. 6b).

We found the primary tumor size did not significantly change with atorvastatin treatment (Fig. 6d). Primary tumors were marked by central regions of necrosis (Fig. 6c). Primary tumor proliferation was quantified by assessing the Ki-67 positivity of the peripheral zone of the tumor. The proliferation of the primary tumor was not significantly affected by atorvastatin treatment (Fig. 6e, f). In contrast to the primary tumor, the lung metastases were much smaller and exhibited no necrotic regions (Fig. 6g). Moreover, we found that atorvastatin reduced the proliferation of lung metastases in a dose-dependent manner, though this trend did not reach statistical significance in the small number of animals challenged (Fig. 6h, i).

To assess the effect of atorvastatin on any site metastasis or primary tumor, we normalized proliferation rates for both the spleen-to-liver (Fig. 5) and mammary fat pad (MFP)-to-lung (Fig. 6) models to their respective vehicle treatments. We found that atorvastatin did not affect the proliferation of the primary tumors (Figure S8A) yet significantly decreased the proliferation of the metastases in a dose-dependent manner (Figure S8B). These data suggest that statins act to reduce metastatic tumor growth (Figure S8C).

## Discussion

Metastatic disease remains challenging to treat and is responsible for the majority of deaths from breast cancer. Metastases are proposed to begin as dormant micrometastases, which may remain quiescent for years to decades before outgrowing to form mortal macrometastases.^3^ Unfortunately, upon detection of the primary tumor, many women already have established dormant micrometastases,^5^ rendering moot strategies to prevent initial dissemination. Thus, therapies that can suppress emergence of these dormant micrometastases would provide a significant mortality benefit to women with breast cancer. Unfortunately, micrometastases are often resistant to standard therapies,^42^ which motivates the implementation of alternative, safe therapies that can block this mortal emergence of dormant tumor cells.

Statins have been shown to decrease mortality but not the incidence of breast cancer, suggesting they interfere with the metastatic cascade and not primary carcinogenesis. We previously demonstrated that statins selectively target cells undergoing EMT, as epithelial cells are relatively more resistant to statin treatment. In this study, we confirm our previous findings that expression of E-cadherin on the membrane of the mesenchymal MDA-MB-231 cells was sufficient to increase resistance to atorvastatin, yet not to the same degree as the naturally E-cadherin high MCF-7 cells (Fig. 2). Upon western blot and immunofluorescent examination, the MDA-MB-231 Ecad cells showed lower E-cadherin expression and less membrane presentation than MCF-7 cells, which may explain the former’s higher statin susceptibility (Fig. 2). We have previously found autocrine EGFR signaling in the MDA-MB-231 cells forces E-cadherin internalization, resulting in primarily intracellular E-cadherin^2^ unless this autocrine signaling is also abrogated; this underlies the lower level of membrane expression as compared to MCF-7. Moreover, mixed culture of the epithelial MCF-7 and mesenchymal MDA-MB-231 cell lines in the presence of atorvastatin results in a dose-dependent enrichment of the former by atorvastatin selectively suppressing growth of the latter.

To determine the effects of statins in the context of the metastatic microenvironment, we employed in vitro and ex vivo models of breast cancer metastasis to the liver. First, we co-cultured primary human hepatocytes with either epithelial or mesenchymal RFP-labeled breast cancer cells, and probed the influence of atorvastatin and rosuvastatin on hepatic toxicity, cancer cell proliferation, and tumor burden. Importantly, we used relatively small numbers of tumor cells (1000 or 5000) as compared to hepatocytes (6 × 10^5^) in order to replicate micrometastases. As previously stated, co-culture with hepatocytes is sufficient to upregulate E-cadherin expression in the MDA-MB-231 cells.^2,40^ We found that rosuvastatin but not atorvastatin was toxic to the hepatocytes at high doses, with a reduction in nuclei number of 40% (Fig. 3). The hepatotoxicity of rosuvastatin has been reported in the literature and is thought to be due to the higher selective uptake of rosuvastatin by hepatocytes as compared to other statins.^43^ When probing cancer cell proliferation, we found that the epithelial MCF-7 cells were relatively less proliferative than the mesenchymal MDA-MB-231 cells at baseline. Moreover, MCF-7 proliferation was unaffected by atorvastatin therapy whereas MDA-MB-231 proliferation was significantly suppressed by atorvastatin in a dose-dependent manner (Fig. 3). These data suggest the susceptibility of these cell lines to atorvastatin-mediated growth suppression is maintained in the context of the hepatocyte microenvironment.

When we compared atorvastatin and rosuvastatin efficacy in MDA-MB-231 cells, we found that rosuvastatin was significantly less potent, despite it having a higher in vitro affinity for its target enzyme, HMGCR, than atorvastatin.^44^ While the lipophilic atorvastatin can diffuse across tumor cell membranes, the hydrophilic rosuvastatin relies more on active transport. Hepatocytes but not breast cancer cells express the OATP1B1 and 1B3 transporters responsible for statin cellular uptake.^45^ We have previously shown that atorvastatin is more effective than rosuvastatin at suppressing cancer cell growth.^24,34^ These data additionally suggest that the direct effects of statins on tumor cells play a larger role in proliferation suppression than indirect effects from statins influencing the neighboring hepatocytes. Moreover, we found atorvastatin could reduce tumor burden—defined as the normalized area of breast cancer cells—at the highest dose whereas rosuvastatin was ineffective (Fig. 3). At the highest dose of 20 µM atorvastatin, normalized RFP area is reduced by 50% whereas proliferation is completely inhibited on the last day of culture (as assessed by EdU). This is likely due to the slow proliferation rate of these cells, particularly in co-culture with hepatocytes. These data also suggest that, in the context of the hepatic microenvironment, statins act mainly in a cytostatic manner, as the anti-proliferation effects occur at lower doses than a reduction in tumor burden (Fig. 3). In support, previous studies have shown statins can induce apoptosis in breast cancer cells in vitro but not in human biopsy samples after neoadjuvant statin treatment in breast cancer patients.^46,47^

Since the PI3K–Akt and MAP kinase signaling networks play important roles in breast cancer proliferation and survival, we investigated whether inhibition of PI3K or Mek1/2 would potentiate atorvastatin in breast cancer co-culture with primary human hepatocytes. While hepatocyte health and tumor burden were unaffected by PI3K or Mek1/2 inhibition, we found that PI3K but not Mek1/2 could potentiate the anti-proliferative effects of atorvastatin (Fig. 4). Namely, we found a significant interaction between PI3K inhibition and atorvastatin treatment (*p* = 0.01) which was not seen with Mek1/2 inhibition (Fig. 4). These data suggest that the PI3K–Akt pathway is important for cellular response to atorvastatin therapy. This would be consistent with atorvastatin diminishing Ras prenylation and its attachment to intracellular membranes.^24^

To investigate the effects of atorvastatin on dormant micrometastasis outgrowth, we employed an all-human microphysiological system of breast cancer metastasis to the liver.^30–33^ After enriching the dormant breast cancer cell population with doxorubicin treatment, we treated these hepatic-breast cancer microtissues with atorvastatin for 96 h. During the last 48 h of treatment, we stimulated with an LPS/EGF stimulus, which is similar to inflammatory signals that enter the liver through portal venous system.^32^ We found that atorvastatin treatment significantly decreased LPS/EGF stimulated outgrowth of the mesenchymal MDA-MB-231 cells. These data suggest that atorvastatin can suppress emergence of the statin-sensitive MDA-MB-231 cells from tumor dormancy.

In order to determine the effect of atorvastatin on the breast cancer metastatic cascade, we employed two independent models of spontaneous breast cancer metastasis: spleen-to-liver and MFP-to-lung. Importantly, these models encompass the entire metastatic cascade, beginning with growth of a primary tumor and ending with distant metastases. This is in contrast to tail vein or intra-cardiac injection models, which primarily probe direct hematogenous seeding of cancer cells and bypass the pathophysiologic changes needed for both initial escape from the primary tumor and seeding as singular cells in the ectopic tissues. In our spleen-to-liver metastasis model, we found that atorvastatin treatment could significantly suppress proliferation of liver metastatic nodules but not the primary tumor (Fig. 5). Moreover, this effect on proliferation was dose dependent. Atorvastatin did not induce cell death in either the primary tumor or metastatic nodules, confirming our in vitro evidence that statins mainly act as growth suppressive in the context of the tumor microenvironment. In our MFP-to-lung metastasis model, we found that atorvastatin did not affect primary tumor proliferation but reduced proliferation of lung metastases (Fig. 6). We believe the atorvastatin-mediated suppression of lung metastasis proliferation did not achieve statistical significance due to a higher variability in spontaneous metastasis to the lung, which can be observed in vehicle treated mice. Importantly, when we normalized and combined proliferation rates for both of our models, we found that atorvastatin significantly suppressed metastasis proliferation but had no effect on primary tumor proliferation (Figure S8).

These in vivo effects are significant for several reasons. First, the doses of atorvastatin used are similar to those used for moderate-intensity lipid lowering therapy in clinical patients.^48^ Accounting for the difference in volume of distribution, the 2 mg/kg and 10 mg/kg doses in mice are approximately equivalent to 20 mg and 80 mg doses in human patients.^49^ Thus, the doses of atorvastatin that suppress metastatic proliferation are similar to those already employed clinically with little systemic toxicity. Second, we found a divergent effect of atorvastatin on breast cancer cells. While the primary tumor cells were unaffected by statin treatment, the metastatic cells were suppressed. This was found in our two mouse models and in our MPS model of breast cancer metastasis to the liver. These results are similar to other reports that statins can delay the formation of metastases in mice.^50,51^ Breast cancer cells are known to re-express E-cadherin and enter a period of quiescence at the metastatic site.^2^ Since atorvastatin was able to suppress stimulated outgrowth of dormant MDA-MB-231 cells, this suggests that atorvastatin can suppress emergence of dormant tumor cells.

The divergent effects of atorvastatin on the primary and metastatic tumor cells is supported by the clinical data suggesting a mortality but not incidence benefit of statins in breast cancer.^10,11^ Other studies have reported statin suppression of primary tumor growth and induction of apoptosis in mouse models.^16,52^ However, previous studies have not compared proliferation at the primary and metastatic sites and used doses higher than those used for cholesterol lowering. Our data suggest atorvastatin can preferentially suppress metastatic breast cancer outgrowth and as such, may be useful as a long-term adjuvant for preventing emergence of dormant breast cancer micrometastases that eventually progress to clinically evident disease. This effect on the emergent mesenchymal tumor cells but not the primary site mesenchymal tumor cells presents a conundrum. It may be that statins are particularly effective in suppressing the transition between the epithelial-like and mesenchymal-like state, or that a higher density of mesenchymal cancer cells, achievable in 3D but not 2D, present a sufficient concentration of autocrine factors to overcome the deficiency of proliferative signaling caused by limited prenylation.^24^ The molecular mechanism for this selectivity remains for future investigations. One limitation of this work is the use of intraperitoneal drug delivery. While this delivery method was chosen to best control the amount of drug used, statins are administered orally to human patients. Since the concentrations needed to achieve these anti-metastasis effects are currently seen with clinical use in cardiovascular disease, targeted clinical trials of adjuvant statin therapy in breast cancer patients at risk for metastatic recurrence may improve mortality without causing significant toxicity. Moreover, atorvastatin efficacy should be examined in metastasis models of other cancer types, such as metastatic prostate cancer.

## Electronic supplementary material

Supplemental Figure 1

Supplemental Figure 2

Supplemental Figure 3

Supplemental Figure 4

Supplemental Figure 5

Supplemental Figure 6

Supplemental Figure 7

Supplemental Figure 8

Supporting Information Legends

## Availability of data and materials

All data not included in this manuscript or the supplementary materials will be available upon request.
