# Supplementary figures and images for: Statins attenuate outgrowth of breast cancer metastases
Source: Br J Cancer. 2018 Nov 7;119(9):1094–105. doi: 10.1038/s41416-018-0267-7 (PMC6220112; doi:10.1038/s41416-018-0267-7)

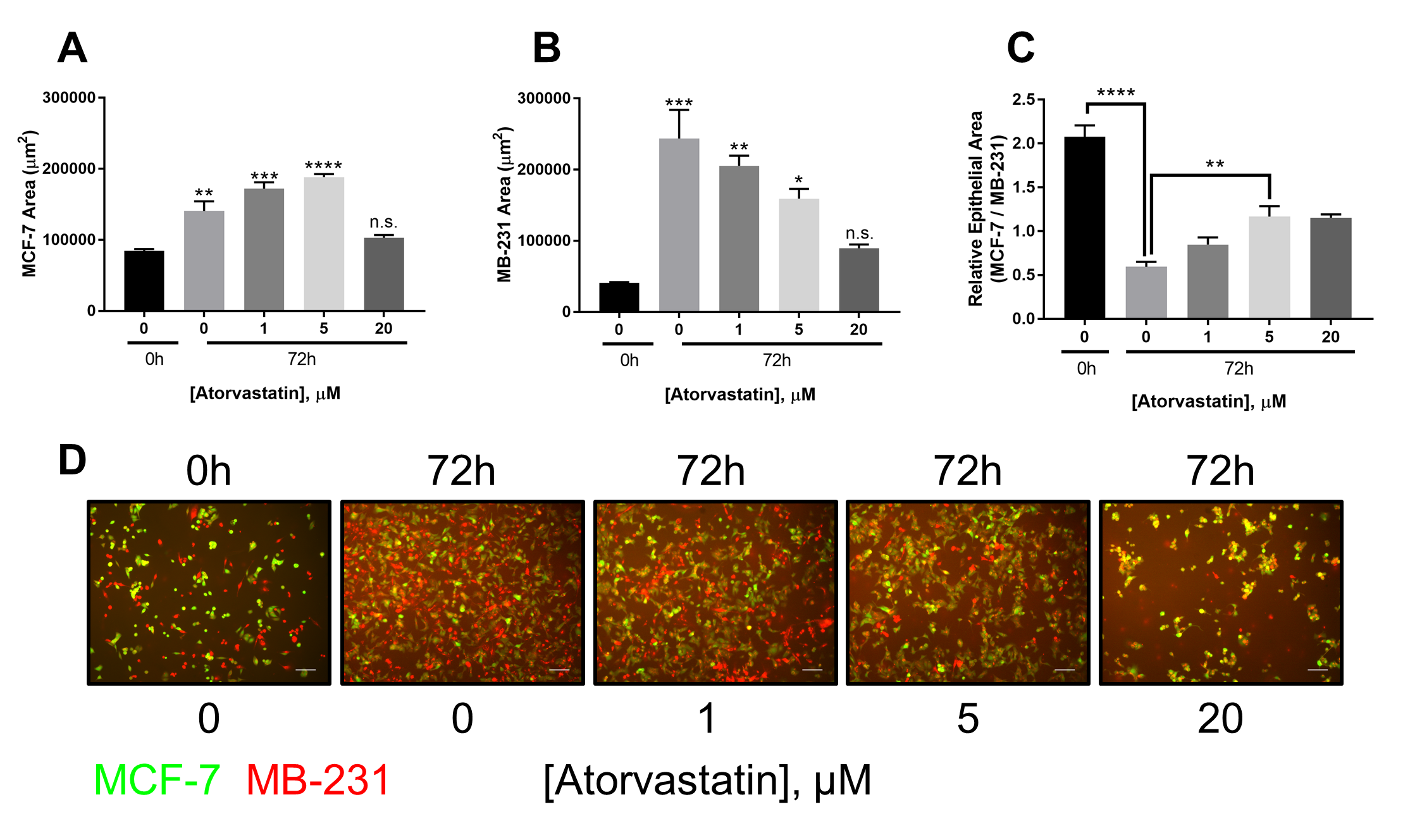

Supplement: Supplementary file 1 — Supplemental Figure 1 [file 41416_2018_267_MOESM1_ESM.tif]

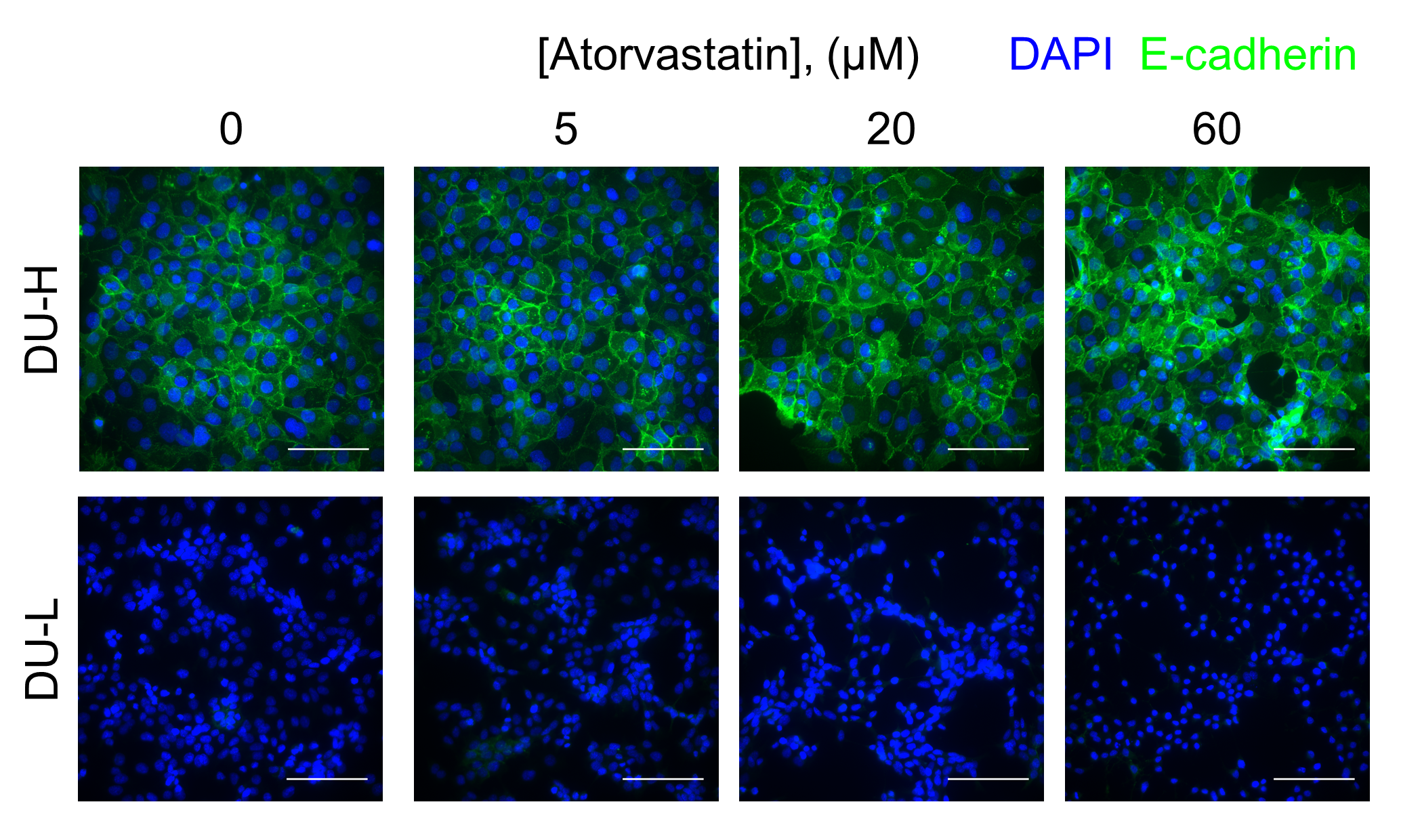

Supplement: Supplementary file 2 — Supplemental Figure 2 [file 41416_2018_267_MOESM2_ESM.tif]

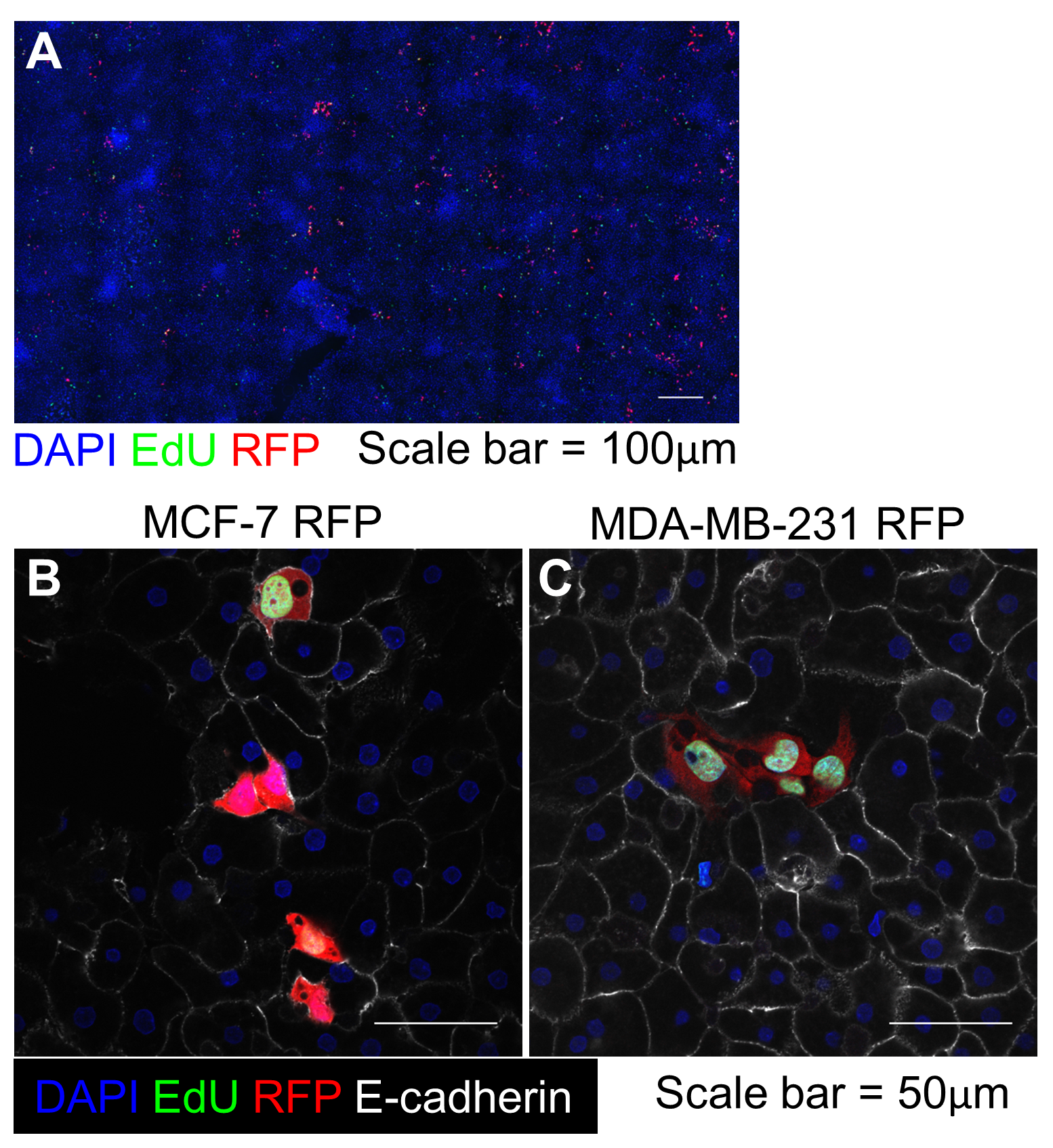

Supplement: Supplementary file 3 — Supplemental Figure 3 [file 41416_2018_267_MOESM3_ESM.tif]

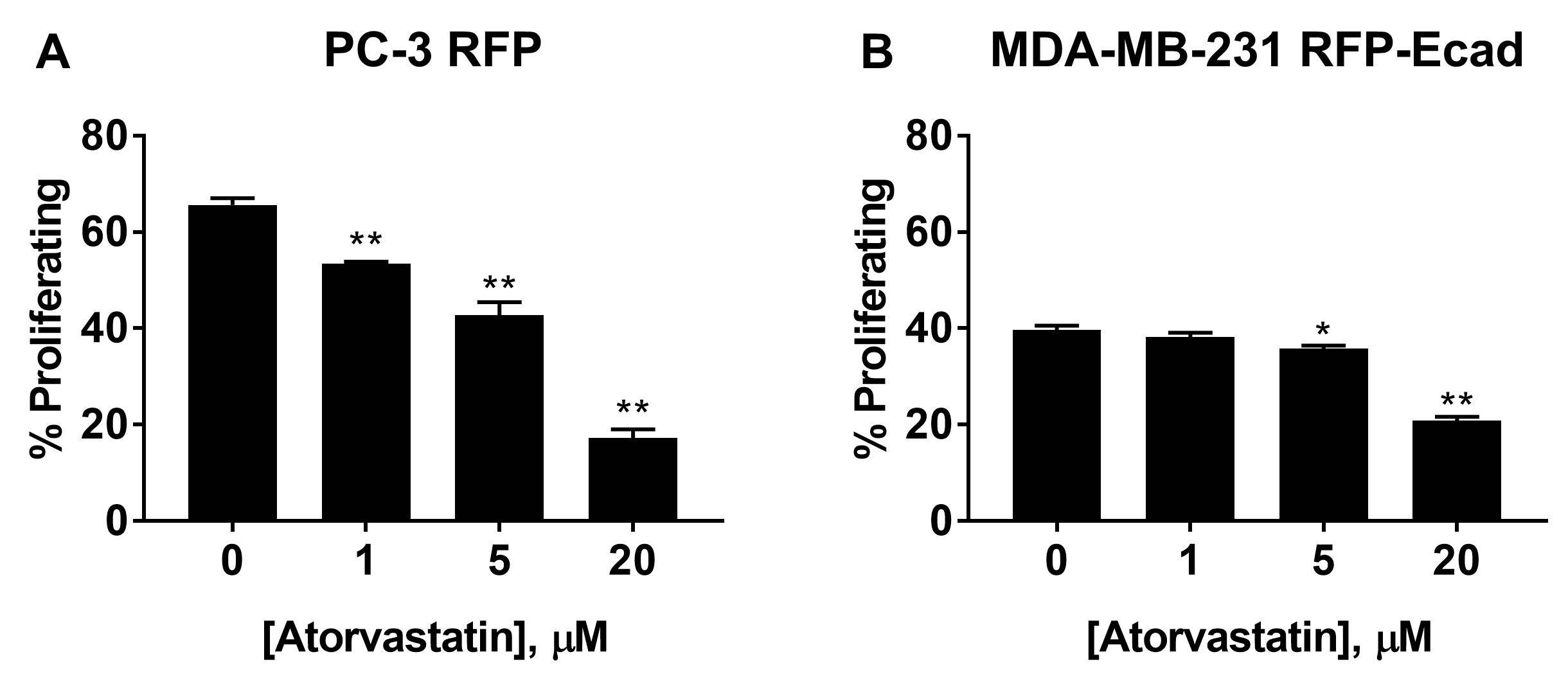

Supplement: Supplementary file 4 — Supplemental Figure 4 [file 41416_2018_267_MOESM4_ESM.tif]

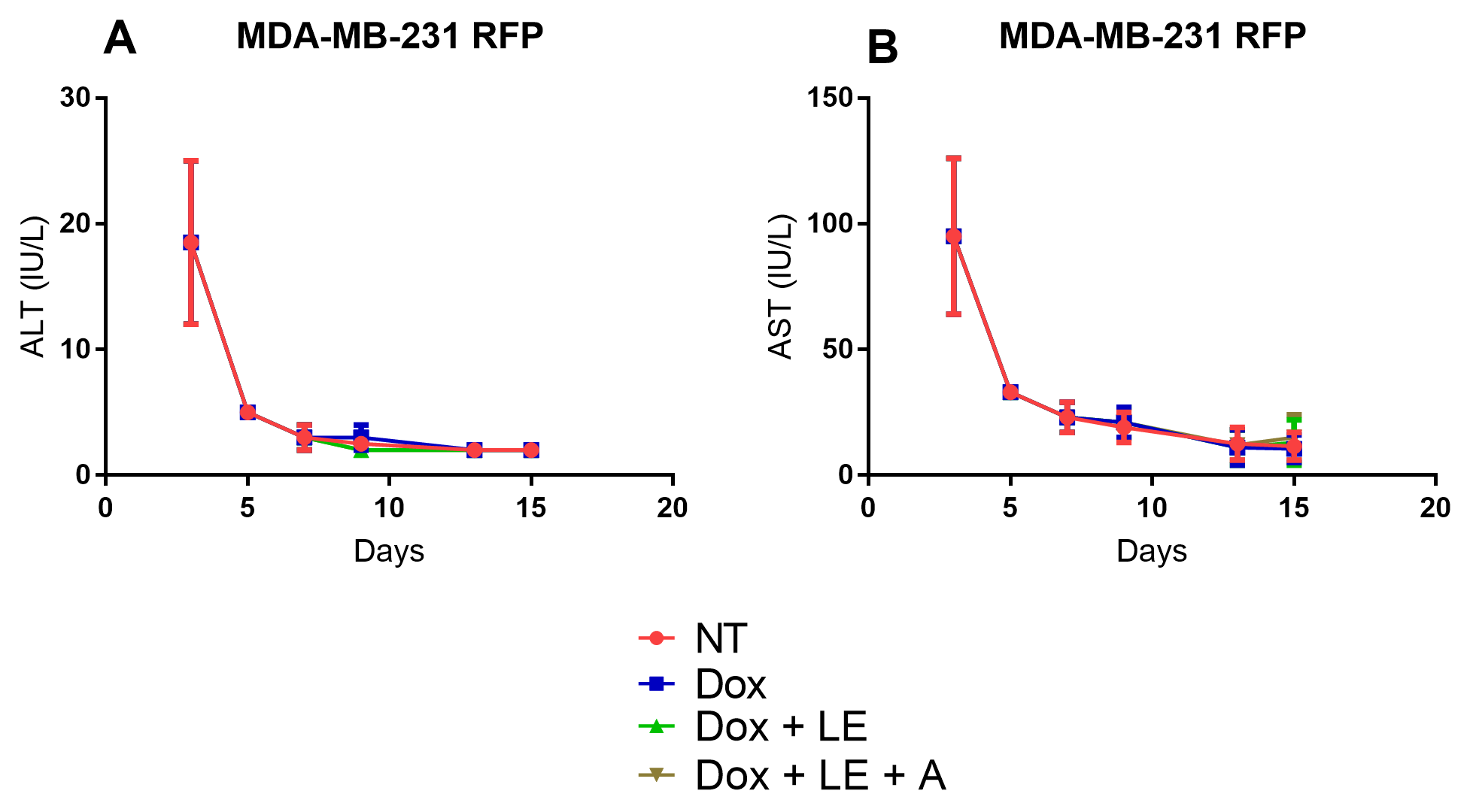

Supplement: Supplementary file 5 — Supplemental Figure 5 [file 41416_2018_267_MOESM5_ESM.tif]

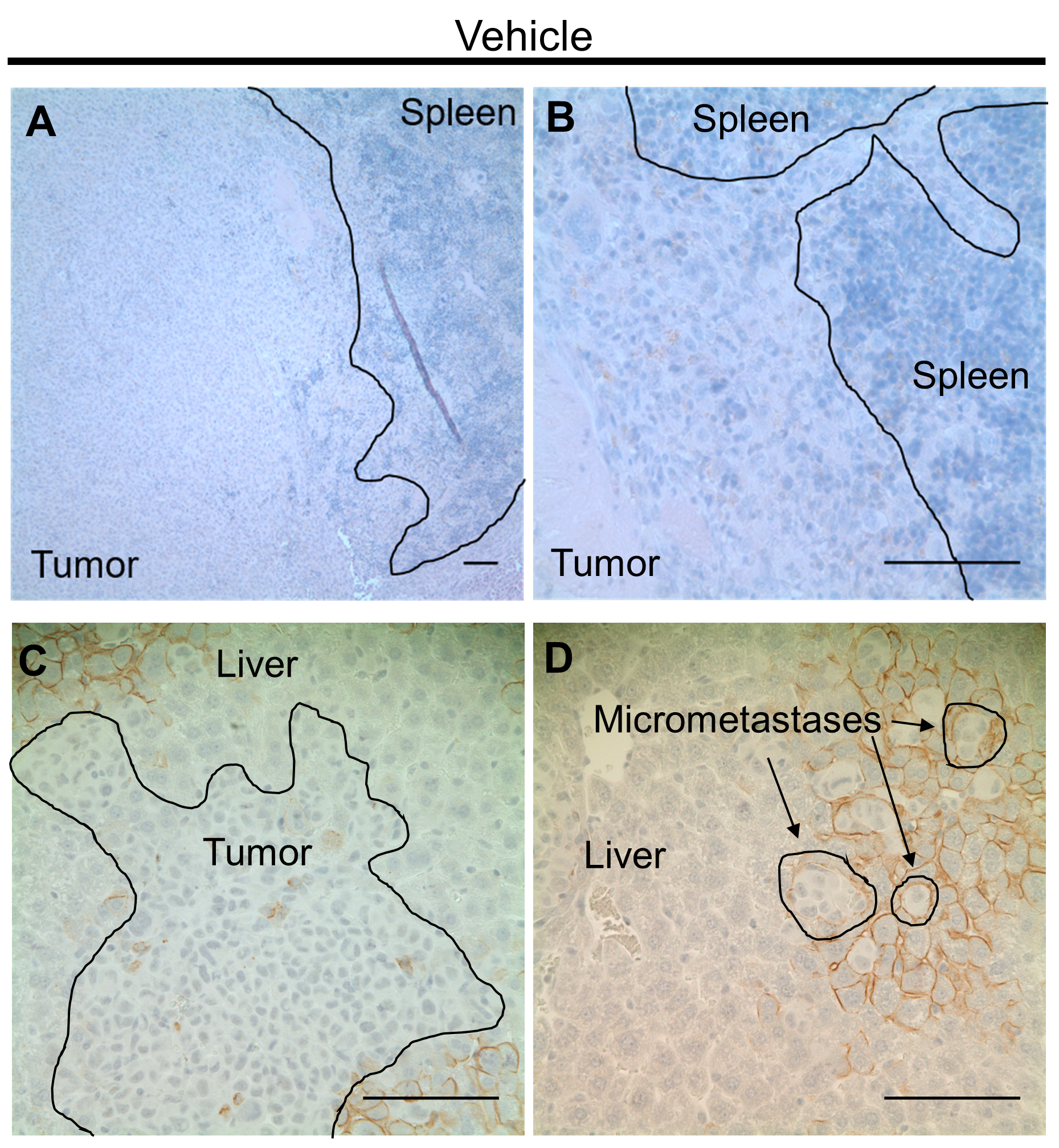

Supplement: Supplementary file 6 — Supplemental Figure 6 [file 41416_2018_267_MOESM6_ESM.tif]

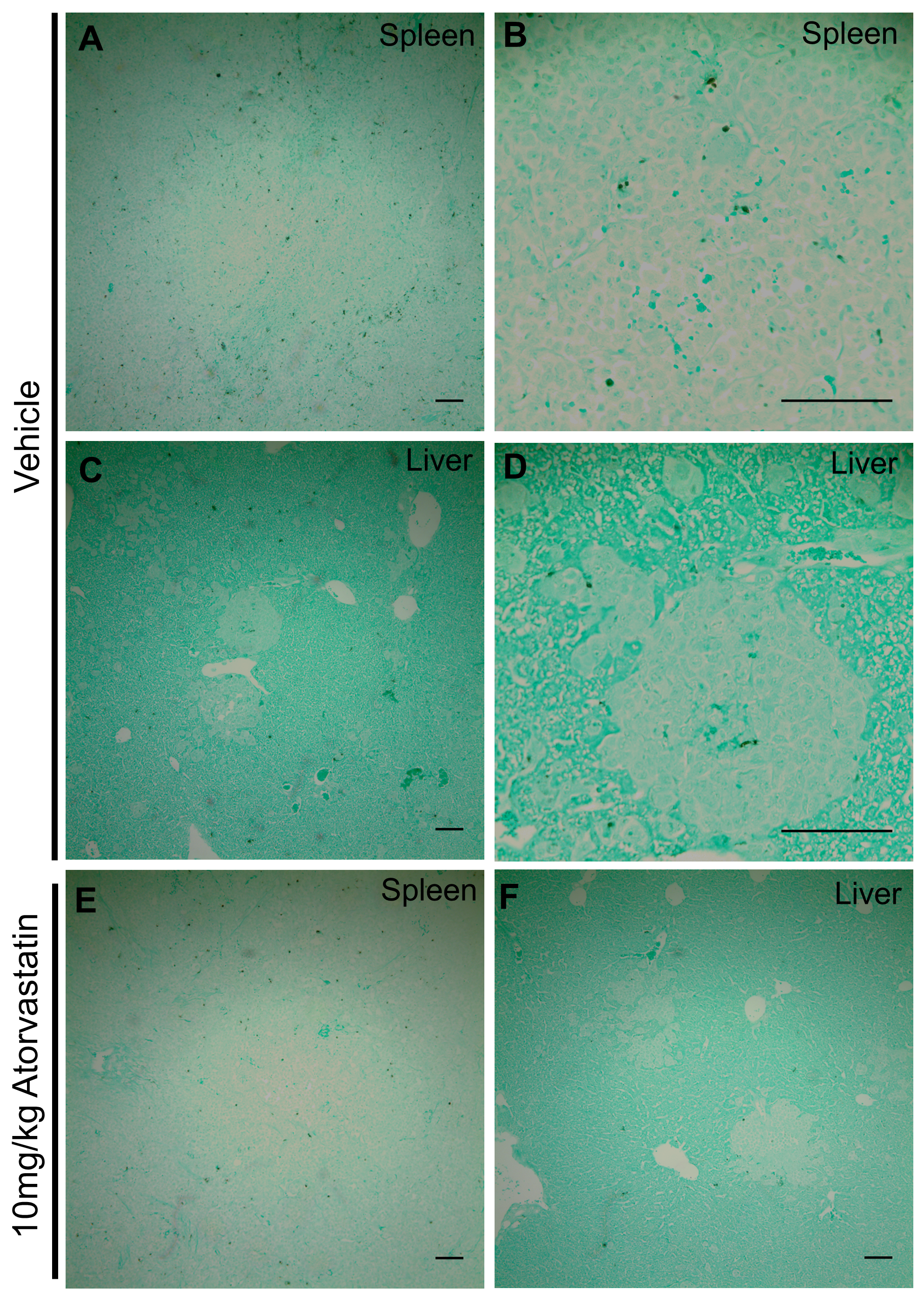

Supplement: Supplementary file 7 — Supplemental Figure 7 [file 41416_2018_267_MOESM7_ESM.tif]

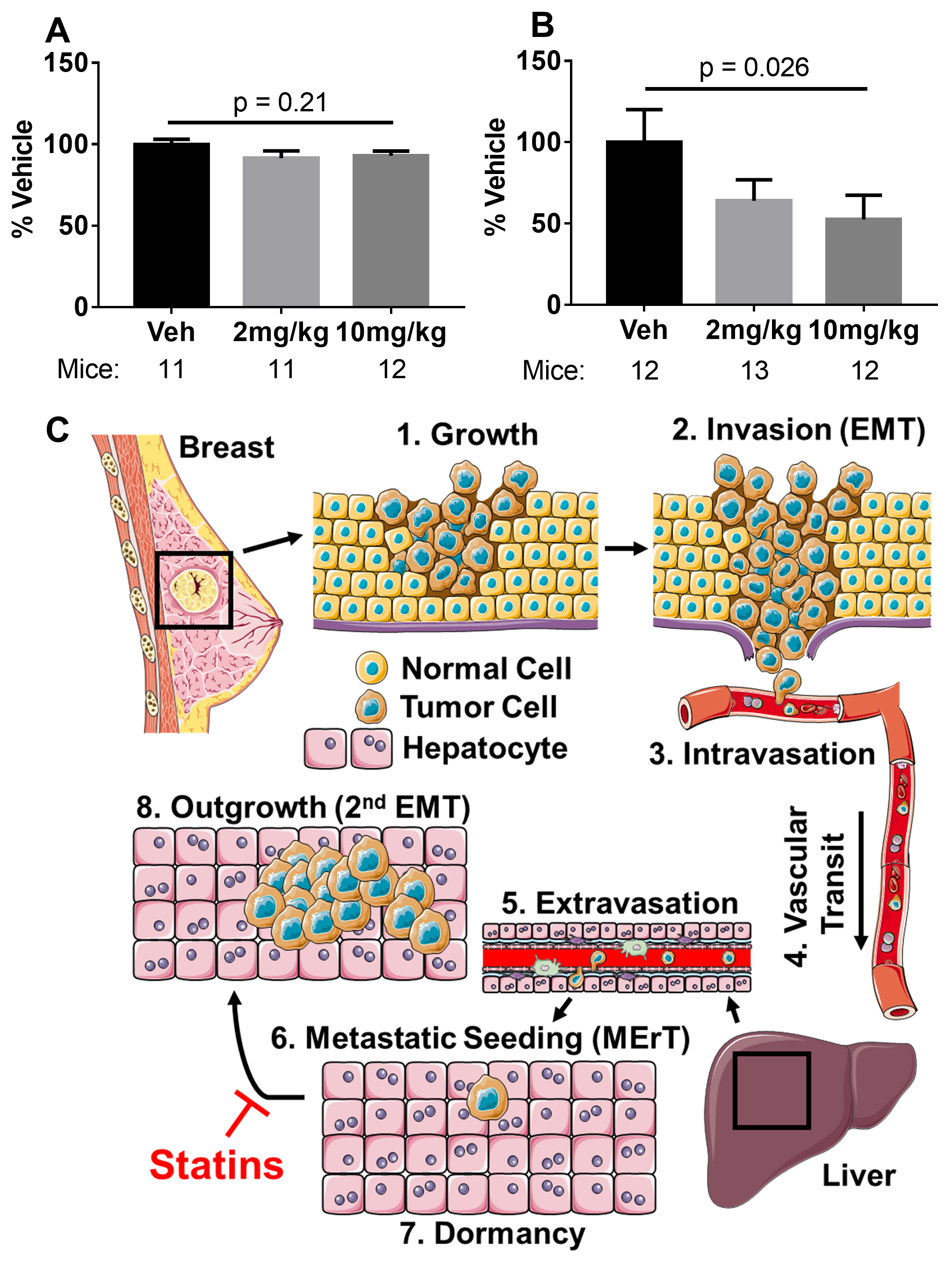

Supplement: Supplementary file 8 — Supplemental Figure 8 [file 41416_2018_267_MOESM8_ESM.tif]
