# Supplementary material for: Statins attenuate outgrowth of breast cancer metastases
Source: Br J Cancer. 2018 Nov 7;119(9):1094–105. doi: 10.1038/s41416-018-0267-7 (PMC6220112; doi:10.1038/s41416-018-0267-7)
Supplement: Supplementary file 9 — Supporting Information Legends [file 41416_2018_267_MOESM9_ESM.docx]

**Supporting Information Legends**

**Figure S1: Mesenchymal breast cancer cells selectively suppressed by atorvastatin.** Mixed cultures of labeled MCF-7 (green, epithelial) and MB-231 (red, mesenchymal) cells were treated with 1µM, 5µM, or 20µM atorvastatin for 72 hours, with 0.04% DMSO serving as the 0µM atorvastatin (vehicle) treatment. The total area of MCF-7 (A), MB-231 (B), and the MCF-7 / MB-231 ratio (C) were calculated using thresholding. Treatment with atorvastatin increased the relative abundance of the epithelial MCF-7 cells by more selectively suppressing growth of the mesenchymal MB-231 cells. Representative images are shown for pre-treatment and after 72 hours of atorvastatin treatment (D). Scale bar = 100µm. * p<0.05, ** p<0.01, *** p<0.001, **** p<0.0001, compared to 0µM atorvastatin treatment for 0 hours unless otherwise indicated; n.s. = not significant.

**Figure S2: Atorvastatin does not change E-cadherin expression in low- and high-expressing DU-145 variants.** DU-H (high E-cadherin) and DU-L (low E-cadherin) cells were cultured for 48 hours in the presence of 5µM, 20µM, or 60µM atorvastatin, with 0.12% DMSO serving as the 0µM atorvastatin (vehicle) treatment. Treatment with atorvastatin did not influence E-cadherin expression in either DU-H or DU-L cells. Scale bar = 100µm.

**Figure S3: Breast cancer and hepatocyte co-cultures.** MDA-MB-231 RFP cells (1000) and MCF-7 RFP (5000) cells were cultured with 6x10^5^ primary human hepatocytes and fixed after 96 hours of co-culture. A widefield microscope was used to take and stitch 140 congruent fields of view (A). Scale bar = 100µm. MCF-7 RFP (B) and MDA-MB-231 RFP (C) co-cultures were additionally stained for E-cadherin, to demonstrate cancer cell integration into the hepatocyte monolayer. Scale bar = 50µm. Blue = DAPI, Green = EdU, Red = RFP, White = E-cadherin.

**Figure S4: Hepatocyte co-cultures with PC-3 RFP and MDA-MB-231 RFP/Ecad.** PC-3 RFP cells (2000) or MDA-MB-231 RFP/Ecad cells (1000) were each cultured with 6x10^5^ primary human hepatocytes and fixed after 96 hours of co-culture. The percentage of proliferating cancer cells were quantified for PC-3 RFP (A) and MDA-MB-231 RFP/Ecad (B) cells. Error bars represent the standard error of the mean (n=3). * p < 0.05, ** p < 0.01 compared to vehicle.

**Figure S5: Atorvastatin does not affect hepatocyte health in the MPS**. Supernatant from MPS wells containing hepatocyte and non-parenchymal cells cultured with MDA-MB-231 RFP were tested for leakage of ALT (A) and AST (B) using assays performed in clinical chemistry laboratories at the University of Pittsburgh Medical Center (UPMC). The initially high markers of hepatocyte damage reflect the isolation protocols. NT = no treatment, Dox = 1µM doxorubicin for days 7-10, LE = 1µg/mL LPS + 20ng/mL EGF for days 13-15, and A = 5µM atorvastatin for days 11-15. Error bars represent the standard error of the mean (n=2).

**Figure S6: MDA-MB-231 RFP cells re-express E-cadherin in small liver micrometastases**. MDA-MB-231 RFP cells in the intrasplenic inoculation model of breast cancer metastasis to liver (Figure 5) were assessed for E-cadherin expression in the splenic primary tumor (A,B) and large (C) or small (D) liver metastases. Scale bar = 100µm

**Figure S7: Atorvastatin does not cause death of MDA-MB-231 RFP primary tumor or metastatic cells**. MDA-MB-231 RFP cells in the intrasplenic inoculation model of breast cancer metastasis to liver (Figure 5) were assessed for cell death by TUNEL staining. Cell death was assessed for vehicle (A-D) and 10mg/kg atorvastatin (E-F) treatment in the splenic primary tumor (A,B,E) and liver metastases (C,D,F). Scale bar = 100µm

**Figure S8: Atorvastatin suppresses breast cancer metastasis growth but not primary tumor growth**. Proliferation of primary tumor cells and metastatic tumor cells obtained for the intrasplenic inoculation model of spontaneous breast cancer metastasis to liver (Figure 5) and the mammary fat pad (MFP) inoculation model of spontaneous breast cancer metastasis to lung (Figure 6) were normalized to their respective vehicle controls. Primary tumor cell proliferation was assessed by combining the normalized proliferation values for the splenic and MFP experiments (A). Metastatic tumor cell proliferation was assessed by combining the normalized proliferation values for the liver and lung metastases (B). A model for the proposed stage of the breast cancer metastatic cascade in which statins act (C). Error bars represent the standard error of the mean. Panel C was made using open access images, courtesy of Servier Medical Art^53^.
